# Adjusting for informative missing outcome data in clinical trials with longitudinal study designs

**DOI:** 10.1186/1745-6215-14-S1-P109

**Published:** 2013-11-29

**Authors:** Ruwanthi Kolamunnage-Dona, Colin Powel, Paula Williamson

**Affiliations:** 1 Department of Biostatistics, University of Liverpool, Liverpool, UK; 2 School of Medicine, Cardiff University, Cardiff, UK

## 

In many clinical trials with longitudinal outcome data, a common situation is where some patients withdraw or dropout from the trial before completing the measurement schedule. In most cases reasons for dropout is related to the subsequent outcome, hence missingess is informative or non-ignorable. Failure to take appropriate account of such missing data can lead to biased estimation of treatment effects. Imputation methods fail for predicting the missing data, as they rely on assumptions that are unverifiable from the observed data. "Joint modelling" is a novel statistical methodology that can be used to analyse the longitudinal outcome data adjusting for non-ignorable missingness. The methodology combines the information from the longitudinal outcome data and the subsequent dropout patterns over time.

In the MAGNETIC trial (a randomised, placebo controlled trial of nebulised Magnesium Sulphate in acute severe asthma in children) the primary outcome, Yung Asthma Severity Score (ASS), was measured at baseline and 20, 40, 60, 120, 180 and 240 minutes following randomisation. The missingness in ASS outcome at various time points was over 5% and reasons for missingness were sometimes clearly related with study withdrawal due to good or poor status of the child, but in many instances these reasons were not known or unclear. Ignoring the informative nature of the missing outcome data in the statistical analysis could result in inaccurate estimation of treatment effect.

